# Lung Cancer Associated with Neurofibromatosis Type I

**DOI:** 10.1155/2013/869793

**Published:** 2013-02-28

**Authors:** Anastasia Oikonomou, Dimitrios Mikroulis, Paraskevi Mintzopoulou, Lawal Lukman, Panos Prassopoulos

**Affiliations:** ^1^Department of Radiology, University Hospital of Alexandroupolis, Democritus University of Thrace, 68100 Alexandroupolis, Greece; ^2^Department of Cardiothoracic Surgery, University Hospital of Alexandroupolis, Democritus University of Thrace, 68100 Alexandroupolis, Greece

## Abstract

Lung cancer associated with neurofibromatosis type I is considered very rare, and only a few case reports have been described in the literature. There is some evidence that a genetic linkage between neurofibromatosis and carcinogenesis in the lung may exist. We present a 42-year-old female, lifetime nonsmoker with a known history of neurofibromatosis type I, free of respiratory symptoms, who underwent a low-dose HRCT of the lungs to investigate any occult interstitial lung changes. A solitary ill-defined nodule of a ground-glass opacity was detected incidentally in the middle lobe with no associated lymphadenopathy or metastatic disease. Several thin-walled lung cysts were also seen in the lower lobes. Histological analysis of the nodule after middle lobectomy revealed well-differentiated adenocarcinoma. The patient did not receive systemic chemotherapy or radiotherapy. She was free of disease on 18-month followup.

## 1. Case Presentation

**Figure 1:**
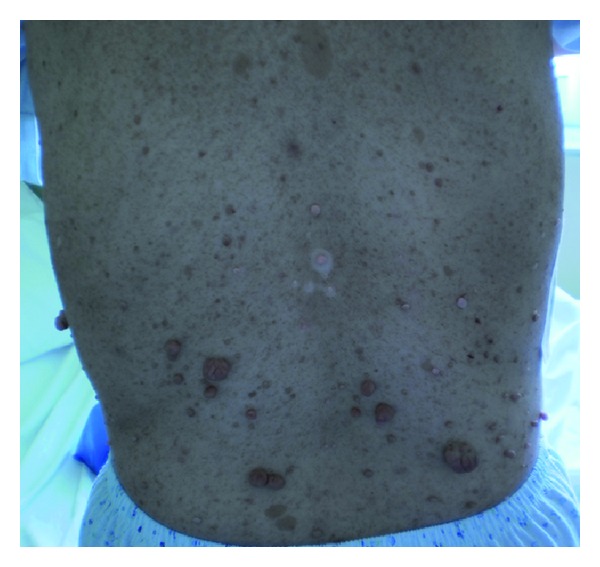
Multiple café-au-lait spots and cutaneous neurofibromas of variable size were noted throughout the patient's body.

A 42-year-old female, lifetime nonsmoker with a known history of neurofibromatosis type I (NF-1) presented to the Outpatient Neurology Clinic for a regular followup. Physical examination revealed multiple café-au-lait spots and cutaneous neurofibromas of variable size throughout her body (Figure 1). She also suffered from vitiligo since the age of 20 years old. Her neurologic examination was unremarkable, and she was free of respiratory symptoms. She reported no history of environmental or occupational exposure to other potential carcinogens for lung cancer. However, as part of her thorough followup, she underwent a low-dose HRCT of the lungs to investigate any occult interstitial lung abnormalities and presence of lung cysts that have been described in neurofibromatosis patients [1].

**Figure 2:**
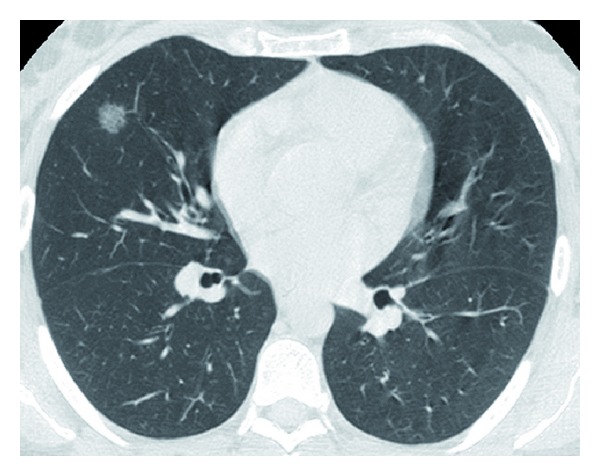
HRCT at the level of the middle lobe shows a 1.4 cm ill-defined nodule of a ground-glass opacity in the right middle lobe demonstrating air alveologram.

HRCT of the lungs detected an incidental ill-defined solitary pulmonary nodule 1.4 cm in diameter, exhibiting a ground-glass opacity and air alveologram (Figure 2). No other lung nodules or areas of consolidation were seen in any other lobes. Several small thin-walled lung cysts were also detected in both the upper and the middle lobes, some of which had a subpleural location. No other interstitial lung changes were seen. There were no enlarged mediastinal or hilar lymph nodes.

Differential diagnosis of the solitary ground-glass nodule included primary lung cancer and an intrapulmonary neurinoma that could be related to neurofibromatosis [2]. 

The patient underwent right anterolateral thoracotomy with a muscle sparing, and after the lung was adequately mobilized, a 1.5 cm nodule was palpated in the middle lobe, which was wedge resected and sent for frozen section. The result was positive for malignancy, and, subsequently, a middle lobectomy and mediastinal lymph node dissection were undertaken. Histological analysis of the nodule revealed well-differentiated minimally invasive lung adenocarcinoma. The mediastinal lymph nodes were negative for malignancy. Lung cancer was classified as stage IA disease, and no further chemotherapy or radiotherapy was administered according to the guidelines [3]. The patient was free of disease on 18-month followup.

## 2. Discussion

Neurofibromatosis type 1 or von Recklinghausen's disease is an autosomal dominant dysplasia of the ectoderm and mesoderm characterized mainly by the presence of neurofibromas, café-au-lait spots, and pigmented hamartomas in the iris (Lisch nodules) [4]. The NF-1 gene has been localized to chromosome 17q11 and functions as tumor suppressor gene, and its respective gene product has been named as neurofibromin [5]. It has been speculated that the mutation of tumor suppressor NF-1 gene increases the patient's risk for the development of various malignancies mainly derived from the neural crest such as malignant schwannoma, neurofibrosarcoma, intracranial glioma, and pheochromocytoma. However, the association of NF-1 with lung cancer is not common. A review from Japan 20 years ago reported only 11 cases of NF-1 with lung cancer [6], and we interestingly were able to find 7 more cases up to now—without including the presented case—also from the Japanese literature [7–13]. Adenocarcinoma was the most frequent histological diagnosis (72.9%) in the first 11 cases reviewed, while the following 6 cases were diagnosed as nonsmall cell cancer (3 cases) [9, 11, 12], small cell cancer (1 case) [10], poorly differentiated cancer (1 case) [7], adenocarcinoma (1 case) [8], and carcinosarcoma (1 case) [13]. Smoking habit was not always mentioned in the reported cases in the literature, but there were some patients that were never smokers as in our case [8, 13]. In our patient, histology revealed a well-differentiated adenocarcinoma, which is also the most common histological type of lung cancer in nonsmokers, who are by far more common in women [14, 15]. The patient in the presented case was a lifetime nonsmoker and so was her husband. Since there is no association with smoking in the presented case, a question raises whether there is a genetic linkage between NF-1 and the development of lung cancer. NF-1 gene has been mapped in the small part of chromosome 17q [5]. p53 gene belongs to this area and has been implicated in the development of various neoplasms including lung cancer [10]. Moreover, in one study, it has been reported that the loss of heterozygosity in chromosome 17p—but not in 17q—was detected in a patient with small cell carcinoma with NF-1. The authors hypothesized that the inactivation of tumor suppressor gene on chromosome 17p—most likely p53—might have been responsible for the development of small cell lung cancer in that case [10]. However, the association of lung cancer with NF-1 might be coincidental.

The nodule in our patient was of pure ground-glass opacity which is known to have a high probability of malignancy compared to “solid nodules” and represents the “bronchoalveolar” component of lung adenocarcinoma indicating a better prognosis of lung adenocarcinoma [16, 17]. 

It remains to be answered whether the development of lung cancer in this young, never-smoker, female patient with NF-1 simply followed the rules of the epidemiology of lung cancer in never-smokers or there is a genetic link between NF-1 and carcinogenesis in the lung. Large genetic-based studies are needed in order to investigate these intriguing findings. The evaluation of pulmonary involvement in NF-1 patients with low-dose chest CT in long intervals could be proposed in order to detect early development of lung cancer or interstitial lung disease.
